# Acoustic Emission Behavior of Early Age Concrete Monitored by Embedded Sensors

**DOI:** 10.3390/ma7106908

**Published:** 2014-10-02

**Authors:** Lei Qin, Hong-Wei Ren, Bi-Qin Dong, Feng Xing

**Affiliations:** 1 School of Civil Engineering and Architecture, University of Jinan, Jinan 250022, China; 2 School of Electrical Engineering, University of Jinan, Jinan 250022, China; E-Mail: cse_renhw@ujn.edu.cn; 3 School of Civil Engineering, Guangdong Province Key Laboratory of Durability for Marine Civil Engineering, Shenzhen University, Shenzhen 518060, China; E-Mail: xingf@sze.edu.cn

**Keywords:** acoustic emission, piezoelectric composites, early age concrete, mass concrete, cracking

## Abstract

Acoustic emission (AE) is capable of monitoring the cracking activities inside materials. In this study, embedded sensors were employed to monitor the AE behavior of early age concrete. Type 1–3 cement-based piezoelectric composites, which had lower mechanical quality factor and acoustic impedance, were fabricated and used to make sensors. Sensors made of the composites illustrated broadband frequency response. In a laboratory, the cracking of early age concrete was monitored to recognize different hydration stages. The sensors were also embedded in a mass concrete foundation to localize the temperature gradient cracks.

## 1. Introduction

The hardening process of concrete or other cement-based materials is considered to be the most critical time period during the life of a structure. It is important to have reliable information about the early age properties of the materials. Classical techniques, such as thermal analysis, X-ray diffraction (XRD) and scanning electron microscopy, have been often used [1]. These characterizations are required to be done *ex situ* after stopping hydration [2,3].

In the past 20 years, *in situ* characterizing techniques for early age cement-based materials have been developed and some of them were based on ultrasonic wave measurement [4,5,6,7,8,9]. In most cases, ultrasonic transducers were coupled onto the mold full of fresh cement-based material and ultrasonic waves were emitted from one side and received on the opposite side. The full hardening process could be continuously monitored. Chotard *et al.* [10] used acoustic emission (AE), which is a passive ultrasonic technique, to characterize the hydration process of calcium aluminate cement. It was found that the AE activity was closely related to the internal change in the cement paste and different period could be characterized.

AE is defined as the class of phenomena whereby transient elastic waves are generated by the rapid release of energy from localized sources within a material or structure. It could provide an instantaneous response to crack growth inside a structure member and it is capable of determining the location of cracking [11]. In civil engineering, the AE technique is mainly applied to concrete, which is the most widely used cement-based material. The AE technique is hard to be used for early-age concrete because AE sensors are mainly designed for hardened concrete. Chotard *et al.* [10] had to use protruding bars, which were used to introduce the signal to the AE sensors. This method is very inconvenient for *in situ* application.

If AE sensors are designed to be easily embedded into fresh concrete, it will be very convenient for early-age concrete. Recently, piezoelectric type sensors suitable to be embedded into concrete were designed and used for monitoring cement hydration, but not used as AE sensors for crack monitoring [12,13]. The AE sensor should have broadband frequency response for cracking detection [11,14]. To fabricate a broadband sensor, the piezoelectric element should have matched acoustic impedance with concrete and a mechanic quality factor as low as possible. Thus, the wave energy in the piezoelectric element would disperse into surrounded medium as soon as possible, and the sensor has flat and wide frequency response. However, the piezoelectric ceramic, which is the most widely used smart material, has very high mechanical quality factor and acoustic impedance. It is difficult to fabricate broadband AE sensors using piezoelectric ceramics for early age concrete. It was reported that piezoelectric composites made of active piezoelectric ceramic and passive material, such as epoxy, could be tailored to meet certain requirement by designing connectivity and adjusting its volume fraction in different phases [15,16,17]. Recently, cement-based piezoelectric composites have been developed. Cement-based piezoelectric composites, named Type 1–3 composites, were proposed for special use in concrete for their compatibility with concrete [18,19].

In this study, Type 1–3 composites were fabricated and used for embedded sensors in concrete to detect cracking during hydration and hardening. The properties of the piezoelectric composites and the frequency response of the sensors were tested. In a laboratory, the hydration process was monitored by embedded and surface bonded AE sensors. Embedded AE sensors were also used in a mass concrete foundation of a high-rise building to detect the inside cracking. In mass concrete, a steep thermal gradient within the concrete could arise due to the hydration heat of cement and due to heat transfer, thus, causing the concrete structure to be exposed to the risk of thermal cracking when subjected to external or internal restraints [20]. Therefore, estimating the existing thermal stresses and the thermal cracks in concrete structures is vital. In the foundation, thermocouples were also embedded into concrete to obtain the temperature gradient development. The AE sensors were used to detect and localize cracks. The relationship between temperature gradient and crack development was analyzed.

## 2. Piezoelectric Composites and Material Properties

**Figure 1:**
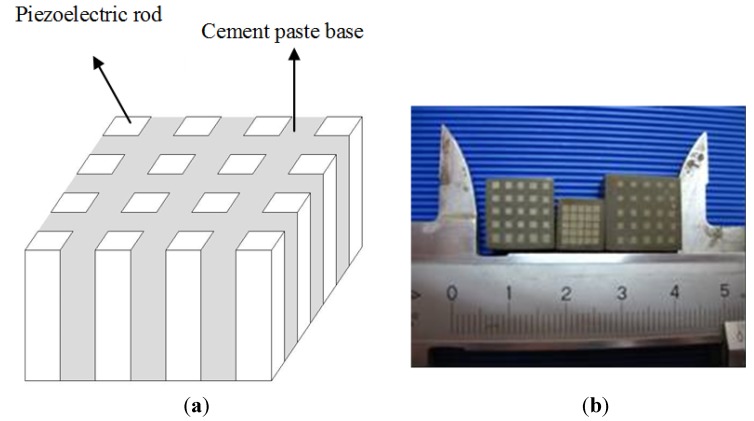
Schematic representation of Type 1–3 cement-based piezoelectric composites. (**a**) schematic representation of Type 1–3 piezoelectric composites; (**b**) picture of the composites of different volume fractions.

**Figure 2:**
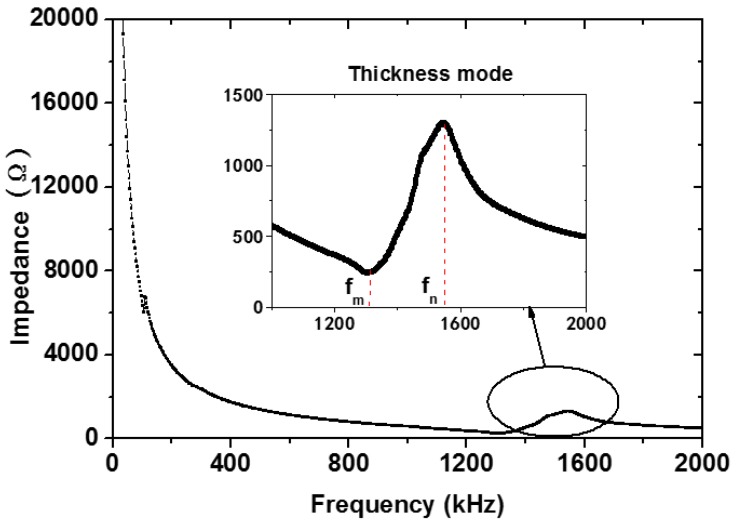
The impedance spectrum of Type 1–3 cement-based piezoelectric composites.

The piezoelectric composites were fabricated by cutting and filling technique. First, a series of piezoelectric ceramic rods were cut along the polarization direction of the piezoelectric ceramic block. During cutting, a common base of the block was left to hold all the ceramic rods in it. According to previous research, the ceramic volume fraction was about 35%. Cement paste (*w*/*c* ratio = 0.45) was cast into the ceramic rods [19]. Then, it was put into a curing box and cured for seven days. The composites are shown in Figure 1. After curing, a thin plate of 1.5 mm was cut off the composites. The plate was adequately dried, and then silver paint was painted onto the opposite surfaces of the plate as electrodes for property measurement. Mechanical quality factor and acoustic impedance were mainly investigated. Mechanical quality factor Q*_m_* could be calculated as follows [21]:

(1) $${\text{Q}}_{m}\text{=}\frac{1}{2\pi f_{s}RC\text{(}f_{p}^{2}\text{-}f_{s}^{2}\text{)/}f_{p}^{2}}$$

where *f_s_* and *f_p_* are series and parallel resonance frequency, which can be approximately replaced by frequency *f_m_* and *f_n_*, which are the frequency at minimum and maximum electric impedance, respectively. *f_m_* and *f_n_* were measured using Agilent 4294A Impedance Phase Analyzer (Agilent, Santa Clara, CA, USA). The impedance spectrum was shown in Figure 2. The window of thickness mode spectrum gives the value of the *f_m_* and *f_n_*. *R* is the minimum impedance value. The capacitance *C* was also measured by the Phase Analyzer at 1 kHz.

The acoustic impedance of the composites *Z* is:

(2) Z=ρv

where ρ is the density of the composites, and *v* is wave velocity of the composites, which could be calculated as *v* = 2*f_s_t*. *t* is the thickness of the composite plate. Q*_m_* and *Z* for the piezoelectric ceramic used in the test are 27.7 and 35.3 Mrayl. The Q*_m_* of the composites is only 7.7, showing a very obvious decrease. The acoustic impedance of the composites is 10.4 Mrayl, near the value of concrete.

## 3. Piezoelectric Sensor Fabrication and Its Frequency Response

The coaxial wire was connected to the electrodes of the composite plate and then enclosed by mixture made of epoxy and cement powder. The mixture was tuned to have a similar acoustic impedance to the piezo-composites by adjusting the cement/epoxy ratio to 3.5 (in weight). The sensors made of the cement-based composites (Type 1–3 sensor) in this research were all 20 × 20 × 20 mm.

**Figure 3:**
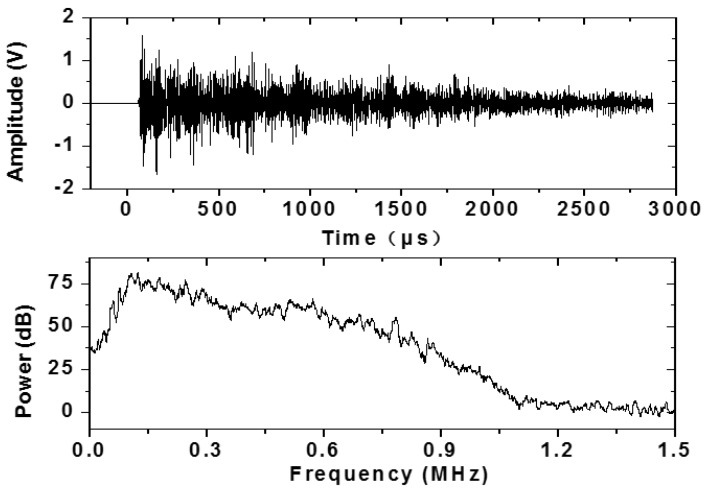
The waveform and frequency spectrum of the signal (Upper part is the wave in time domain and the lower is wave in frequency domain).

To obtain the frequency response of the sensor, the sensor was bonded onto an aluminum plate (1000 × 1000 × 50 mm) using silica grease, 100 mm away from the AE source. In this characterization, a pencil with the Nielson Shoe was used to break 0.5 mm 2H lead to generate acoustic waves [22]. The waves were recorded and analyzed by the PCI-2 AE system from Physical Acoustic CORP. (PAC). The response of the Type 1–3 sensor is shown in Figure 3. It could be seen that the sensor has a relatively flat response over the range of 50 to 600 kHz, and did not drop off until about 700 kHz. The broadband frequency response of the sensor could meet the AE monitoring requirement of concrete material.

## 4. AE Monitoring of Early Age Concrete in a Laboratory

**Figure 4:**
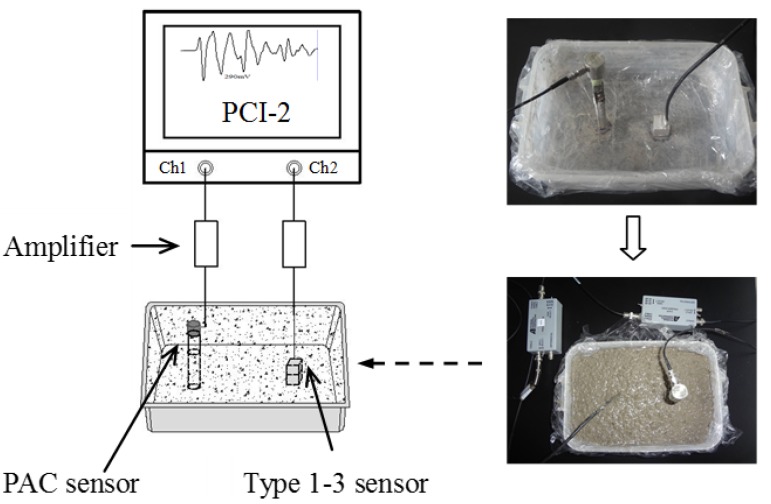
Instruments used for early age concrete AE monitoring in laboratory.

The test instrument is shown in Figure 4. In the test, a plastic box (200 × 100 × 50 mm) was used as mold to hold the fresh concrete. Type 1–3 sensor was attached onto a 20-mm-thick plate, which was bonded to the bottom of the mold. A steel bar was vertically bonded onto the bottom of the mold and a PAC AE sensor (WSa model) was attached onto the top of the bar. The PAC sensor had an operation frequency range of 100–1000 kHz. A layer of cling wrap was attached to the inner side of the mold walls to avoid the AE signals from the interface between the concrete and the mold wall, which might be induced by the contraction of the concrete. The acoustic emission system is a PCI-2 system. Two sensors were connected to the system through two amplifiers. During AE monitoring, the temperature was also measured using a waterproof thermocouple embedded in the center of a similar mold, full of fresh concrete. The thermocouple had an accuracy of ±0.1 °C and was calibrated using a normal thermometer in water.

The mixture proportion of fresh concrete and raw materials were all provided by a commercial concrete mixing plant, which supplies concrete for the later mentioned project. The weight of cement, water, sand, aggregate and fly ash were 310, 202, 695, 1157 and 133 kg/m^3^ respectively. Water reducer was also admixed and the dosage was 0.0215% of cement in weight. P O 32.5 Portland cement was used and the maximum size of the course aggregate was 20 mm. Because the concrete was designed for usage in mass concrete, fly ash was added to reduce the hydration heat. After mixing, the mixture was vibrated to eliminate air bubbles and then the concrete was poured into the plastic mold. During testing, the specimen molds were put into a big moisture plastic box acting as a curing room and the room temperature was about 20 °C.

**Figure 5:**
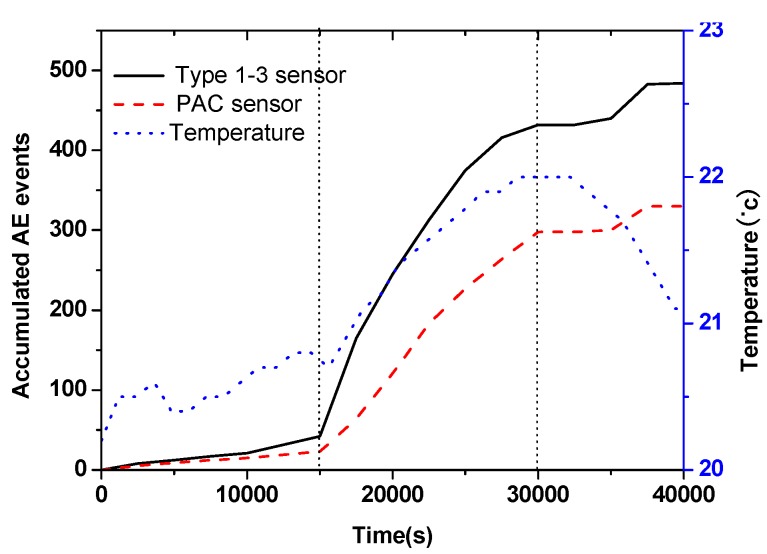
The accumulated AE event and temperature development during hardening of the concrete.

**Figure 6:**
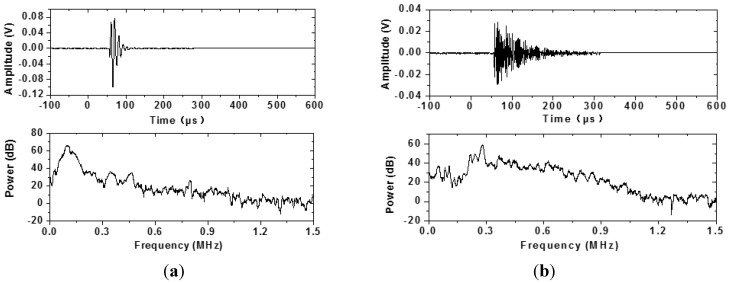
The AE signals recorded in Stage 2. (**a**) AE signal recorded by Type 1–3 sensor; (**b**) AE signal recorded by PAC sensor.

**Figure 7:**
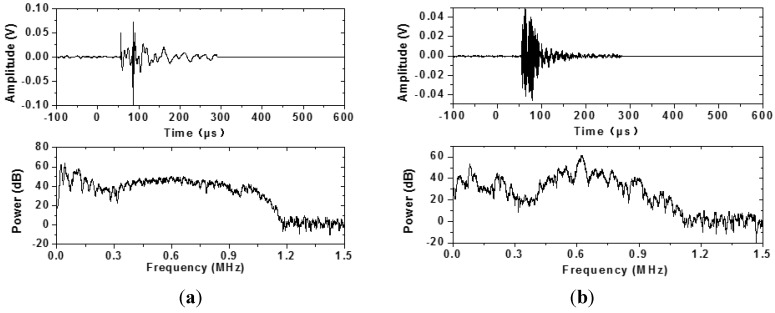
The AE signals recorded in Stage 3. (**a**) AE signal recorded by Type 1–3 sensor; (**b**) AE signal recorded by PAC sensor.

Prior to AE data acquisition, the two sensors were tested to obtain the noise level. During test the threshold was set 15 dB above the noise level. The AE monitoring was carried out continuously. In Figure 5, the accumulated AE event number and temperature development are shown. The developing trend of the accumulated AE signals recorded by the two sensors was very similar. However, more signals were recorded by Type 1–3 sensors because the embedded sensor had better contact with concrete. According to the curves, the hardening process could be divided into three stages. During Stage 1, almost no AE events were recorded because of the minor microstructure development and high attenuation of fresh concrete. The AE events increased very quickly during Stage 2. The quick temperature increase in this stage illustrated the intense hydration and the shrinkage of the concrete-induced the active cracking. During Stage 3, only small amount of AE events were recorded and the temperature began to decrease. More difference could be seen if looking into details of the signals. In Figure 6 and Figure 7, signals that occurred in Stages 2 and 3, but recorded simultaneously by two sensors, were shown in time domain and frequency domain. In the time domain, the wave recorded by the Type 1–3 sensor had a shorter duration time. This was because the AE signal recorded by the PAC sensor had experienced many reflections in the steel bar. In Figure 6, the peak frequency of the wave received by the Type 1–3 sensor was about 100 kHz. The peak frequency of the wave recorded by the PAC sensor was about 300 kHz. In Figure 7, the power of high frequency wave increased obviously. In Figure 7a, the power between 300 and 900 kHz reached about 40 dB. The peak frequency of the wave was about 600 kHz in Figure 7b. In Stage 3, the AE signals should have a higher peak frequency because of the increase of modulus. Additionally, the higher frequency wave should pass the concrete with low attenuation more easily.

## 5. Temperature Gradient Crack Localization for Mass Concrete

**Figure 8:**
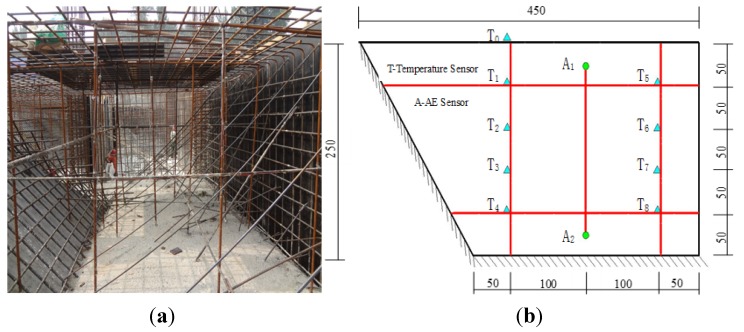
The arrangement of the temperature and AE sensors in the mass concrete. (**a**) the picture of the foundation during construction; (**b**) the location of the sensors and the unit is cm).

The foundation is the base element of a 100-m high-rise building in Jinan city of Shandong province, China. The depth of the foundation is 2.5 m. According to the Chinese code, JGJ3-2010, the foundation belongs to a mass concrete specimen and the temperature of the foundation at early age should be monitored [23]. The concrete was supplied from a concrete plant and the same as the concrete tested in aforementioned section. Thermocouples were embedded into the foundation before casting concrete and connected to a temperature auto-recording system. Type 1–3 sensors were also embedded to monitor the cracking activity. The mass foundation and location of the sensors are shown in Figure 8. The temperature sensors were bonded on the side of steel bars using adhesive tape and the distance between the temperature sensors was 50 cm. The AE sensors were coupled to the top and bottom ends of the steel bars using epoxy, and the distance between the AE sensors was 200 cm. AE activity was recorded and the AE source was localized using PCI-2 system. It was assumed that the cracking occurred on the surface of the steel bar. The localization can be deduced using the differences among the first wave arrival times at each of the AE sensors. This application is a typical one-dimension case. The localization mechanism is illustrated as:

(3) $$\Delta t=t_{2}-t_{1}=\left|x_{2}-x_{s}\right|/\text{υ}-\left|x_{1}-x_{s}\right|/\text{υ}$$

where ∆*t* represents the difference of arrival time between sensor 1 and 2; *x_s_* is the coordinate of the AE source to be determined; *x*_1_ and *x*_2_ are the coordinates of the sensors; υ is the wave velocity in steel and the value is 5200 m/s. Obtaining the difference of the arrival time, the AE source could be easily determined.

**Figure 9:**
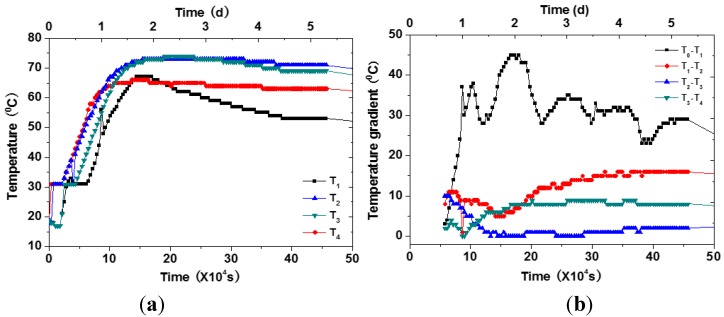
The temperature and gradient development. (**a**) Temperature development; (**b**) temperature gradient development.

The development of temperature is shown in Figure 9. At the very beginning, the temperature inside concrete increased very quickly. On the second day, the temperature reached the peak point. Then, temperature decreased very slowly. The temperature gradient between neighbor points is also shown in the figure. The gradient between sensor *T*_1_ and *T*_2_ was very pronounced. The development of the temperature and gradient for temperature sensors 5–8 were very similar.

**Figure 10:**
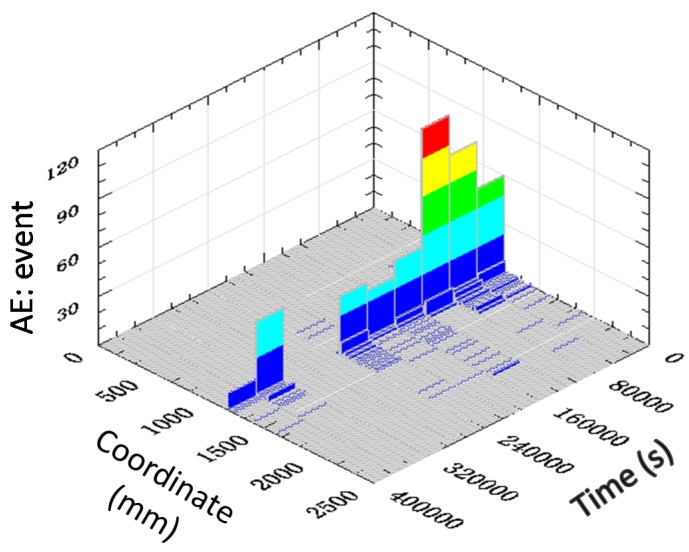
The location and AE event number due to temperature gradient in mass concrete.

Cracking would occur when the tensile stress caused by the accumulation of temperature gradient exceeds the tensile strength of concrete. The locations of cracking inside the mass of concrete were determined by PCI-2 system using the method illustrated in Equation (3). The coordinates of two AE sensors were 250 and 2250 mm. These AE signals, which were localized between the two sensors, were valid ones. The information of the AE event number and crack location is shown in Figure 10. The three axes of the figure are the AE event number, crack location, and time, respectively. Prior to about 10 × 10^4^ s (about 28 h), a large amount of cracking was recorded and, after that point, cracking continued, but with a lower amount per unit time. It could also be seen that most cracks occurred at or above the middle of the depth. This could be related to the temperature gradient development shown in Figure 9b. The major gradient happened in the upper part of the foundation and reached its first peak at about 10 × 10^4^ s. Thus, the cracks recorded in this case were mainly induced by the temperature gradient.

## 6. Conclusions

In order to obtain the cracking information of concrete at early age, a broadband AE sensor was developed based on the Type 1–3 cement-based piezoelectric composites. The composites had a lower mechanical quality factor and acoustic impedance. The sensor made of Type 1–3 composites showed considerable broadband frequency response. In a laboratory, AE sensors were used to record cracking during the hydration process. Three stages could be divided according to the accumulated AE numbers. Most AE signals induced by the shrinkage of specimen were recorded during the intense hydration period. In the mass concrete foundation, the temperature gradient cracking was localized by the embedded AE sensors. The significance of the Type 1–3 sensor was shown in the *in situ* AE monitoring for concrete material and structures.
